# Do Different Interdependencies within a Sport Affect the Perceived Motivational Climate, Use of Spontaneous Self-Talk, Positivity or Precompetitive Anxiety?

**DOI:** 10.5114/jhk/204776

**Published:** 2025-09-23

**Authors:** Luis Arturo Gómez-Landero, Francisco Javier Santos-Rosa, Xoana Reguera-López-de-la-Osa, Cervelló Eduardo

**Affiliations:** 1 Physical Performance & Sports Research Center, Department of Sports and Computer Science, Universidad Pablo de Olavide, Seville, Spain.; 2 Education, Physical Activity and Health Research Group, Department of Special Didactics, University of Vigo, Vigo, Spain.; 3 Sport Research Centre, Department of Sport Sciences, University Miguel Hernández of Elche, Elche, Spain.

**Keywords:** training, competition, team sport, rhythmic gymnastics, individual sport

## Abstract

Comparative studies often find conflicting psychological differences between individual and team sports, likely due to mixing sports with different levels of interdependence and structures. To address this issue, this study focused on the same sport, comparing individual rhythmic (IR) and team rhythmic (TR) gymnastics modalities. The study aimed to assess differences in the perceived motivational climate, self-talk, positivity, and precompetitive anxiety between these two groups, and to identify psychological predictors of precompetitive anxiety. Seventy-six senior female gymnasts were divided into IR (n = 41, aged 15.41 ± 2.47 years) and TR (n = 35, aged 15.22 ± 2.59 years) groups. Results showed that TR gymnasts perceived a greater ego-involved climate, indicating higher intra-team rivalry, and used more positive self-talk in training, leading to better confidence and concentration. However, no significant differences in overall positivity were found. IR gymnasts increased their use of positive self-talk from training to competition, whereas TR gymnasts remained consistent. Self-confidence was lower in IR gymnasts, though both groups had similar levels of somatic and cognitive anxiety. While IR gymnasts showed more associations with competitive anxiety, the predictors of anxiety were the same for both groups: negative self-talk during training and positivity. In conclusion, the study highlights the role of positivity and self-talk in managing competitive anxiety, with a focus on controlling team rivalry in TR gymnastics and promoting self-confidence and positive self-talk in IR gymnastics specially during training. The study recommends grouping sports by interdependence and structures to better understand psychological differences between individual and team sports.

## Introduction

Sports have traditionally been categorized into two types based on the degree of interdependence inherent in their tasks: individual and team sports with independence or interdependence tasks, respectively (Evans et al., 2012). However, this dichotomy does not fully account for the varied structures and internal logics observed (Menezes-Fagundes et al., 2021). For example, in team sports such as regattas, the crew must navigate in a variable physical environment. In others, the environment is stable, but with opponents and teammates sharing the same playing area (e.g., basketball) or using two separate playing fields (e.g., volleyball). In contrast, other team sports, such as artistic swimming and aesthetic gymnastics (Armada Martínez et al., 2021) do not involve a direct opposition or a variable physical environment, focusing on performing routines synchronized to musical accompaniment. Thus, this dichotomous classification could be insufficient, since all environments within these categories are not homogenous, limiting the potential to create tailored group-based strategies aimed at improving performance, commitment, and emotional well-being in specific sport settings (Evans et al., 2012). Indeed, structures and perceptions of interdependence could predict athletes' perceptions of cohesion, satisfaction, and competence with their teammates (Evans and Eys, 2015).

Sports such as artistic swimming, figure skating, and rhythmic gymnastics feature both individual and team modalities. Applying the Sport Team Interdependence Typology (Evans et al., 2012) to rhythmic gymnastics, the gymnast performs solitary and independently, without interaction with teammates in the individual modality. Conversely, in the group modality, five gymnasts collaborate as a team performing integrated tasks (Evans et al., 2012), sometimes with coactive dependence (Carron and Chelladurai, 1981) performing individual tasks symmetrically, and other tasks that involve reactive-proactive dependence (Carron and Chelladurai, 1981) such as coordinated throwing and catching. Thus, within the modalities of the same sport, a similar internal structure and logic are maintained, sharing the same motor content (F.I.G., 2017), but they are differentiated by the presence or absence of interdependence among teammates. A comparative analysis of these modalities could provide valuable insights into the effects of interdependence versus independence.

Numerous studies have explored and compared individual versus team sports, frequently identifying differences in psychological characteristics and cognitive processes that can influence performance. Regarding coaching styles, athletes in individual sports generally prefer a democratic approach, where the coach involves them in the decision-making process (Aleksic-Veljkovic et al., 2016). Differences have also been observed in personality traits. Individual athletes tend to score higher in conscientiousness, autonomy (Nia and Besharat, 2010), and positive trait-like individual differences (PTLIDs) such as perseverance, positivity, resilience, self-esteem, and self-efficacy (Laborde et al., 2016). In contrast, team athletes exhibit higher levels of agreeableness, sociotropy (Nia and Besharat, 2010), extraversion (Allen et al., 2013; Singh, 2017) and openness (Singh, 2017). Additionally, individual athletes are reported to utilize more mental imagery, self-talk, goal setting, and relaxation techniques during practice (Soflu et al., 2011), and are more likely to use goal setting and self-talk in competitive contexts (Ismail, 2019). Conversely, team athletes demonstrate lower levels of negative thinking compared to individual athletes (Soflu et al., 2011).

However, conflicting results have also emerged in the literature. Contrary to many findings, some studies report no significant differences between individual and team athletes in key personality traits. For example, Kemarat et al. (2022) found no differences between the two groups in neuroticism, extraversion, openness to experience, agreeableness, and conscientiousness. Similarly, a comparison of mental toughness and performance strategies, including goal setting, self-talk, attention control, relaxation, emotion regulation, automaticity, confidence, control, and constancy, revealed no significant differences between the two groups (Bayköse et al., 2021). Regarding the perceived motivational climate in individual and team sports, research has revealed negative and direct correlations between ego-oriented and task-oriented climates, with these relationships being stronger and more distinct in team sports (Castro-Sánchez et al., 2018). However, no significant differences were found between individual and team sports when examining goal orientation during training and competition (van de Pol and Kavussanu, 2012). These conflicting findings may be attributed to the heterogeneity of the sports involved. Comparative studies often include a wide variety of individual sports (e.g., squash, gymnastics, tennis, skating, shooting, taekwondo) and team sports (e.g., basketball, rowing, soccer, volleyball, artistic swimming), which differ greatly in nature.

The close relationship between anxiety and sports performance is well-established in the scientific literature, examined from various perspectives. This relationship is relevant in rhythmic gymnastics, characterized by its high demands for precision, body coordination, and apparatus manipulation. Minor errors, such as a slight loss of balance or a fall during competition, can significantly impact the final score and exacerbate perceived anxiety levels (Nassib et al., 2017).

There is evidence of how the perception of the motivational climate influences anxiety. Van de Pol and Kavussanu (2012) emphasized the importance of distinguishing between individual and team sports when investigating motivational climates and related processes in sport. A perceived ego-involving coaching climate has been identified as a predictor of trait anxiety among athletes across both individual and team sports (Armada Martínez et al., 2021; Vazou et al., 2006). Conversely, team athletes who perceived their coaches as fostering a mastery (task)-involving climate experienced reduction in sport-related anxiety (Smith et al., 2007). It has further been suggested that a task-oriented motivational climate may act as a protective factor against anxiety in athletes (Castro-Sánchez et al., 2018), linked with cohesion and optimism (Armada Martínez et al., 2021).

Several studies have highlighted self-talk as a cognitive process associated with competitive anxiety, both during training and in competition (Georgakaki and Karakasidou, 2017; Santos-Rosa et al., 2022). Traditionally, self-talk has been conceptualized as a strategic or deliberate mental tool, without sufficient attention to the more organic or spontaneous self-declarations that emerge in various sporting contexts. Recent definitions now include both goal-directed and spontaneous elements of self-talk, offering a more nuanced understanding of its relationship with performance and performance-related psychological processes (Latinjak et al., 2023; Sarig et al., 2023). Notably, the occurrence of negative spontaneous self-talk has been linked to heightened anxiety levels (Santos-Rosa et al., 2022). The role of individual personality traits in shaping spontaneous self-talk has also been emphasized. For instance, positivity as a personality trait has been found to have a direct association with positive spontaneous self-talk and an indirect relationship with negative self-talk (Santos-Rosa et al., 2022). Additionally, athletes in individual sports tend to display more positive personality traits compared to those in team sports, although these findings do not imply causality (Laborde et al., 2016).

Based on the preceding arguments, questions arise regarding potential differences between individual (independent) and team (interdependent) disciplines within the same sport, which share similar internal structures and logic but differ by the presence or absence of teamwork. Therefore, the present study aimed to (1) examine differences between individual and team modalities in female rhythmic gymnastics with respect to the motivational climate, positivity, spontaneous self-talk (in both training and competition contexts), and anxiety, and (2) identify which psychological variables would predict precompetitive anxiety in both modalities. Given the varying degrees of interdependence between individual and team gymnasts, we anticipated identifying specific differences between these modalities in terms of both the variables under comparison and the predictors of competitive anxiety. Based on existing literature, we proposed the following hypotheses for the first objective:

1H1. Gymnasts aim to replicate a pre-determined gestural model during competition (F.I.G., 2017). In team gymnastics, frequent comparisons among teammates are typical to ensure alignment with the gestural model. Therefore, we expected that the team modality would exhibit higher levels of ego-involving motivational climate compared to the individual modality.

1H2. Based on current evidence (Kemarat et al., 2022; Laborde et al., 2016), we did not anticipate finding significant differences in positivity between individual and team gymnasts.

1H3. We hypothesized that negative self-talk would decrease from training to competition as a form of adaptive response to stress (Sarig et al., 2023) across all gymnasts.

1H4. Considering that individual gymnasts train and compete independently, bearing the full burden of competitive stress (Pluhar et al., 2019), we expected them to experience higher levels of precompetitive anxiety compared to their team counterparts. Specifically, this anxiety was anticipated to manifest as increased cognitive and somatic anxiety, alongside reduced self-confidence.

For the second objective, we hypothesized that:

2H1. The perception of an ego-involving motivational climate would be expected to positively predict both somatic and cognitive anxiety, as supported by previous research (Armada Martínez et al., 2021; Vazou et al., 2006).

2H2. We anticipated that positivity as a personal trait would exhibit predictive capacity regarding precompetitive anxiety, with an indirect relationship with somatic and cognitive anxiety and a direct relationship with self-confidence (Santos-Rosa et al., 2022).

2H3. The frequency of negative self-talk was hypothesized to predict self-confidence indirectly and to have a direct predictive relationship with cognitive and somatic anxiety (Hatzigeorgiadis and Biddle, 2008; Santos-Rosa et al., 2022).

## Methods

### Participants

Previous research has identified the influence of gender (Patsiaouras et al., 2017), the skill level and experience (Madsen et al., 2022) on behavioral and psychological differences among athletes, thus purposive sampling was employed to select participants and address the research questions. All procedures adhered to the principles outlined in the Declaration of Helsinki. Informed consent was obtained from all participants or their parents when minor gymnasts were concerned, following a comprehensive explanation of the study's objectives and methodology. Eligible gymnasts were required to have a minimum of four years of competitive experience and must have participated in national championships in the absolute category. Additionally, they needed to have competed exclusively in either the individual or the team (group) modality for the past two years. Sample size required for the given statistical power was calculated with G*Power Software v.3.1.9.6. To achieve 80% of power (α = 0.05), the minimum required sample size ranged from 36 to 51 for the differences between two independent samples, from 34 to 64 for correlational analysis, and from 42 to 68 for linear multiple regression. Finally, the study sample comprised 76 senior female rhythmic gymnasts from 18 different clubs, aged 15.32 ± 2.51 years. Selected gymnasts trained between 12 and 15 hours per week during the season in which the study was conducted, increasing to 15–18 hours in the period leading up to competitions. They were divided into two groups according to the individual rhythmic (IR, n = 41, aged 15.41 ± 2.47 years) and team rhythmic modality (TR, n = 35, aged 15.22 ± 2.59 years). Both groups showed no differences in age (*p* = 0.61).

### Measures

The reliability of the instruments used was generally classified as good (0.9 > Cronbach’s α ≥ 0.8) for most measures, except for the task-involving climate, positivity, cognitive anxiety, and self-confidence, which were deemed acceptable (0.8 > Cronbach’s α ≥ 0.7) (Sharma, 2016).

### Perception of the Motivational Climate

The Spanish-translated version of the Motivational Climate Perception Questionnaire in Sports-2, as adapted by Balaguer et al. (1997), was used. Second-order factors were examined rated from 1 (strongly disagree) to 5 (strongly agree). The term "player" was replaced by "gymnast" according to the sample used. The Spanish version has shown factorial patterns and internal consistency coefficients similar to those reported by Balaguer et al. (1997).

### Positivity

The Spanish version of the Positivity Scale was employed (Laborde et al., 2016). This scale comprises eight items rated on a Likert scale from 1 (strongly disagree) to 5 (strongly agree), assessing positive views of oneself, one’s life, future, and trust in others. The instrument fitted well and exhibited strong internal consistency in its adaptation to Spanish.

### Spontaneous Self-Talk in Training and Competition

The Spanish version of the Automatic Self-Talk Questionnaire for Sports was employed to assess the content and structure of athletes' self-talk (Latinjak et al., 2016). This instrument evaluates eight dimensions: four positive (concentration, anxiety control, confidence, and instructions) and four negative ones (worry, withdrawal, somatic fatigue, and irrelevant thoughts). The questionnaire was adapted for the context of gymnastics and for two distinct situations (Santos-Rosa et al., 2022): (1) regular training, assessed with items such as “In your sport, how often have you thought or said to yourself something similar to the following ideas in the last few months?”, and (2) competition, assessed with items such as “In this competition, how often have you thought or said something similar to the following ideas?”. Participants indicated their frequency on a Likert scale from 1 (never) to 5 (very often). The instrument has exhibited robust factor structure and internal consistency in both training and competition settings (Santos-Rosa et al., 2022).

### Precompetitive Anxiety

We used the Spanish version of the Competitive Sport Anxiety Inventory 2-Revised (CSAI-2R) (Andrade Fernández et al., 2007). This instrument assesses cognitive state anxiety, somatic state anxiety, and self-confidence within a competitive context. Each item on the CSAI-2R is rated on a Likert scale from 1 (not at all) to 4 (very much). The instrument has shown good fit values in exploratory and confirmatory factor analyses (Santos-Rosa et al., 2022).

### Design and Procedures

This study employed a cross-sectional design, with data collection divided into three phases: training (1), pre-competition (2), and post-competition (3). The procedures were standardized across all participants and clubs to ensure consistency and reliability of the collected data. Explanations and test administration were conducted by the same researchers throughout all phases. The selection of questionnaires and explanation of protocols were reviewed by a collaborator with expertise in sports psychology, ensuring methodological rigor.

(1) Data were collected during a regular training session, excluding competitive contexts. Training times varied between 16:30 and 21:00 across clubs; however, all assessments took place during the hour prior to the last training session of the week (Friday for all clubs). Measures included positivity as a personality trait, perception of the motivational climate, and self-talk during the training situation. Participants completed the questionnaires simultaneously, sitting separately to ensure individual responses.

(2) Data on precompetitive anxiety were collected 15 to 20 minutes before the start of the competition. This phase occurred during two significant tournaments, both qualifying events for the National Championship: one in the individual modality and another in the team modality (groups). Measurements were conducted during the beginning of the official warm-up period prior to the competition routine, assigned to each gymnast or the team.

(3) Measurements of self-talk in competition were obtained within 5 minutes after the performance. Gymnasts (individual or team) completed the questionnaires immediately after leaving the competition area and receiving their official scores, following the protocols of rhythmic gymnastics tournaments. Participants completed the questionnaires individually while sitting separately. No verbal feedback about their performance was provided during this process.

### Statistical Analysis

Data analysis was conducted with JASP Statistics Software v.0.18.0 and Microsoft Excel (2019). Internal consistency reliability of the variables was assessed using Cronbach's alpha, with a threshold of ≥ 0.70 considered acceptable. Descriptive statistics, including means and standard deviations, were calculated for each variable. The Shapiro-Wilk test was employed to evaluate data normality, with statistical significance set at *p* < 0.05.

To compare contextual, personal, and situational factors between groups (IR, TR), independent samples *t*-tests were conducted. For variables with non-normal distributions, the Mann-Whitney U test was used. To analyze differences in self-talk across contexts (training vs. competition), paired-samples *t*-tests or Wilcoxon signed-rank tests were applied depending on the data distribution. Effect size was given by the Cohen's *d* in the *t*-test (ES *d* < 0.20 very small; 0.20–0.49 small; 0.50–0.79 moderate; ≥ 0.80 large), and Glass's rank biserial correlation for Mann-Whitney U tests (ESrb < 0.10 very small; 0.10–0.29 small; 0.30–0.49 moderate; ≥ 0.50 large). The Pearson correlation coefficient was used to detect possible predictors of precompetitive anxiety (i.e., cognitive, somatic, self-confidence), for each group separately. For non-parametric variables, Spearman’s rho was used. The magnitude of correlation coefficients was interpreted according to Hopkins (2006): 0.0–0.09 trivial, 0.1–0.29 small, 0.3–0.49 moderate, 0.5–0.69 large, 0.7–0.89 very large, and 0.9–0.99 nearly perfect. Lastly, stepwise multiple linear regression analysis was conducted to determine predictors of each component of precompetitive anxiety, separated by group. Assumptions for regression analysis included: linearity of relationships with precompetitive anxiety, variance inflation factor < 5, absence of autocorrelation, homoscedasticity, and normality of residuals.

## Results

Several significant differences were observed between groups (Table 1). TR exhibited higher values in the ego-involving climate, positive self-talk in training and self-confidence. In detail, differences were identified in the subscales, with higher scores in intra-team member rivalry (*p* = 0.007, ESrb = 0.36 moderate), concentration (*p* = 0.002, ESrb = 0.413 moderate) and confidence (*p* = 0.018, ES *d* = 0.555 moderate).

**Table 1:** Means, standard deviations, and comparisons between groups.

	Variables	Team Rhythmic			Individual Rhythmic			*p*	ES
		Mean		SD	Mean		SD		
	Age^†^	15.23	±	2.59	15.42	±	2.48	0.61	−0.068
Training	Ego-Involving climate	2.57	±	0.63	2.23	±	0.58	**0.017**	0.564
	Task-Involving climate^†^	4.51	±	0.48	4.35	±	0.52	0.133	0.201
	Positive Self-talk	3.83	±	0.56	3.39	±	0.62	**0.002**	0.735
	Negative Self-talk	2.15	±	0.80	2.35	±	0.60	0.213	−0.289
	Positivity	4.03	±	0.58	3.91	±	0.51	0.327	0.227
Competition	Positive Self-talk	3.84	±	0.75	3.66	±	0.70	0.281	0.25
	Negative Self-talk^†^	1.44	±	0.51	1.36	±	0.46	0.597	0.071
	Somatic Anxiety	2.40	±	0.84	2.44	±	0.60	0.824	−0.052
	Cognitive Anxiety	2.55	±	0.78	2.52	±	0.62	0.816	0.054
	Self-Confidence^†^	3.68	±	0.35	3.29	±	0.55	**0.001**	0.437

† Mann-Whitney test and effect size by the rank biserial correlation. Bold font indicates significant differences

Table 2 illustrates a reduction in the use of negative spontaneous self-talk during competition compared to the training context in both the TR and IR groups, while showing differentiated behaviors in positive self-talk between groups.

**Table 2:** Comparative use of spontaneous self-talk in training and competition situations.

		Training			Competition			*p*	ES
		Mean		SD	Mean		SD		
Team Rhythmic	Positive Self-talk	3.83	±	0.564	3.841	±	0.748	0.898	−0.022
	Concentration	3.989	±	0.762	3.983	±	0.855	0.966	0.007
	Anxiety control	3.786	±	1.139	3.421	±	1.256	**0.011**	0.454
	Confidence	3.743	±	0.856	4.034	±	0.947	0.054	−0.338
	Instructions	3.794	±	0.9	3.84	±	1.029	0.787	−0.046
	Negative Self-talk	2.145	±	0.795	1.442	±	0.512	**< 0.001**	0.993
	Worry^†^	1.678	±	0.71	1.371	±	0.696	**0.003**	0.65
	Retirement^†^	1.857	±	0.904	1.234	±	0.615	**< 0.001**	0.86
	Somatic fatigue	2.651	±	1.087	1.686	±	0.738	**< 0.001**	1.033
	Irrelevant thoughts	2.693	±	1.202	1.521	±	0.784	**< 0.001**	0.832
Individual Rhythmic	Positive Self-talk^†^	3.392	±	0.622	3.66	±	0.702	**0.010**	−0.472
	Concentration^†^	3.307	±	0.919	3.654	±	0.958	**0.017**	−0.469
	Anxiety control	3.427	±	0.922	3.659	±	1.007	0.171	−0.218
	Confidence	3.288	±	0.789	3.712	±	0.873	**0.006**	−0.452
	Instructions	3.551	±	0.84	3.615	±	0.94	0.676	−0.066
	Negative Self-talk^†^	2.347	±	0.604	1.356	±	0.459	**< 0.001**	0.961
	Worry	1.958	±	0.749	1.359	±	0.593	**< 0.001**	0.701
	Retirement^†^	1.961	±	0.837	1.161	±	0.494	**< 0.001**	0.921
	Somatic fatigue	3.117	±	0.963	1.61	±	0.699	**< 0.001**	1.529
	Irrelevant thoughts^†^	2.543	±	0.917	1.274	±	0.432	**< 0.001**	0.992

† Wilcoxon test and effect size by the rank biserial correlation. Bold font indicates significant differences

Correlational analysis revealed several significant associations with precompetitive anxiety (Table 3). The variables most associated with anxiety in both groups were negative self-talk in the training context and positivity. Positive self-talk in the training context only showed associations in IR gymnasts, while during competition it was only associated with self-confidence in TR gymnasts. Negative self-talk during competition was related to cognitive anxiety in both groups.

**Table 3:** Correlational analysis by groups. Motivational climate, positivity and spontaneous self-talk with precompetitive anxiety.

		Team Rhythmic			Individual Rhythmic		
		Somatic Anxiety	Cognitive Anxiety	Self-Confidence^†^	Somatic Anxiety	Cognitive Anxiety	Self-Confidence^†^
Ego-Involving climate		0.202	0.197	0.199	−0.11	−0.071	−0.06
Rivalry between members^†^		0.131	0.034	0.195	−0.332^*^	−0.309^*^	0.119
Task-Involving climate^†^		0.196	0.159	0.055	−0.017	0.123	−0.005
Training	Positive Self-talk	0.035	−0.081	0.268	0.004	−0.315^*^	0.35^*^
	Confidence	−0.179	−0.302	0.43^*^	−0.392^*^	−0347^*^	0.462^**^
	Instructions^†^	0.32	−0.009	0.072	0.089	−0.339^*^	0.223
	Negative Self-talk	0.267	**0.358** ^*^	0.019	0.488^**^	0.403^*^ ^*^	−0.371^*^
	Worry^†^	0.13	0.323	−0.116	0.405^**^	0.347^*^	−0.441^**^
	Somatic fatigue	0.186	0.201	−0.017	0.445^**^	0.399^*^	−0.368^*^
	Irrelevant thoughts	0.449^**^	0.379^*^	0.165	0.181	0.124	−0.072
Positivity		−0.159	−0.192	0.573^**^	−0.356^*^	−0.394^*^	0.42^**^
Competition	Positive Self-talk	0.248	−0.024	0.346^*^	0.083	−0.17	0.147
	Anxiety control^†^	0.142	0.079	0.120	0.36^*^	−0.181	−0.213
	Confidence^†^	−0.081	−0.304	0.492^*^	−0.082	−0.1	0.272
	Instructions^†^	0.395^*^	0.094	0.147	0.146	−0.058	0.056
	Negative Self-talk^†^	0.318	0.369^*^	−0.242	0.291	0.417^**^	−0.15
	Worry^†^	0.027	0.419^*^	−0.331	0.261	0.354^*^	−0.261
	Somatic fatigue^†^	0.318	0.268	−0.276	0.338^*^	0.399^*^	−0.139

† Spearman's Correlations; * p < 0.05; ** p < 0.01; Bold in associations included in the regression model

Finally, regression analysis identified negative self-talk during training and positivity as the only variables that significantly predicted precompetitive anxiety in both groups. Self-confidence was predicted by positivity (TR, adjusted R2 = 0.363, F(1,34) = 20.356, *p* ≤ 0.001; IR, adjusted R2 = 0.155, F(1,40) = 8.363, *p* = 0.006), while cognitive anxiety was predicted by negative self-talk (TR, adjusted R2 = 0,102, F(1,34) = 4.848, *p* = 0.035; IR, adjusted R2 = 0.141, F(1,40) = 7.575, *p* = 0.009). Somatic anxiety was exclusively predicted by negative self-talk in IR gymnasts (adjusted R2 = 0.219, F(1,40) = 12.213, *p* = 0.001).

## Discussion

The current study examined the differences between individual (independent) and team (interdependent) modalities in rhythmic gymnastics, addressing two specific objectives.

The first explored the influence of the practiced modality on various psychological factors, including the motivational climate, positivity, spontaneous self-talk (during training and competition), and anxiety. Our findings revealed significant differences across most of the variables analyzed, depending on the modality practiced, underscoring the influence of interdependence levels.

1H1. was confirmed. The perception of the motivational climate differed between individual and team rhythmic gymnasts. Team gymnasts perceived a higher ego-involved climate, marked by intra-team rivalry, while both groups perceived a stronger task-involving climate overall. This aligns with previous research showing that task-involving climates foster more adaptive behaviors for performance (Ntoumanis and Biddle, 1999), suggesting that both individual and team gymnasts emphasize the learning process over outcomes.

However, some studies offer contradictory results. Van de Pol and Kavussanu (2012) found no significant impact of the sport type on goal orientation (task or ego). Castro-Sánchez et al. (2018) noted that individual athletes in task-involving climates focused on the important role, while team athletes emphasized cooperation. Ego-involving climates, on the other hand, promoted rivalry, especially in individual sports. These discrepancies may result from the different levels of interdependence and internal structures in various sports (Evans et al., 2012).

Thus, rhythmic gymnasts do not directly compete against each other, but strive to perform routines with precision. This contrasts with individual sports like taekwondo, where direct competition could lead to greater rivalry. The rivalry in team rhythmic gymnastics may stem from synchronized actions and frequent comparisons among teammates, often driven by coaches during training. This ego-involved climate, characterized by "coactive dependence" (Carron and Chelladurai, 1981), may explain the stronger perception of rivalry in team gymnasts compared to individuals. In contrast, team sports with "interactive dependence" (Carron and Chelladurai, 1981), such as water polo or basketball, demand different levels of cooperation between teammates, which may generate different perceptions of the motivational climate (Castro-Sánchez et al., 2018; van de Pol and Kavussanu, 2012) compared to sports such as rowing or team rhythmic gymnastics.

1H2. was confirmed, as no significant differences were found in positivity between individual and team gymnasts, matching the results of Kemarat et al. (2022). Laborde et al. (2016) observed higher levels of positivity, along with other personality traits linked to PTLIDs, in athletes compared to non-athletes. However, when comparing individual and team sports, the differences were less pronounced, with slightly higher values observed in individual sports such as archery, badminton, boxing, gymnastics, and weightlifting compared to team sports such as basketball, beach volleyball, and artistic swimming (Laborde et al., 2016). Similarly, Soflu et al. (2011) found a lower incidence of negative thoughts in team sports compared to individual sports.

These findings appear to contradict our results. While other personality traits have been more consistently differentiated, such as greater conscientiousness in individual sports (Allen et al., 2013; Nia and Besharat, 2010), and higher extraversion in team sports (Allen et al., 2013; Singh, 2017), the impact of the type of sport on athletes’ positivity remains unclear (Kemarat et al., 2022). It is also worth reiterating the importance of the variety of sports classified as individual or team in the aforementioned studies, which may contribute to the conflicting results. Moreover, beyond the type of sport practiced, other factors such as perceived parental education could also influence personality traits, as evidenced by observed associations with certain psychological variables and principles related to sportsmanship (Vega-Díaz and González-García, 2025).

1H3. was confirmed, as both TR and IR groups exhibited a clear reduction in all variables of negative self-talk during competition. Additionally, the use of negative self-talk was similar in both modalities across training and competition contexts. This reduction in negative self-talk during competition aligns with a previous study that examined the relationships between stress, the training versus competition situation, and the eight organic categories of self-talk in individual and team athletes (Sarig et al., 2023). These studies concluded that higher stress levels associated with competition led athletes to make a more conscious effort to regulate critical elements of their performance, which may be indicative of the dual processing theory (Sarig et al., 2023). Therefore, our findings confirm a comparable adaptive response in TR and IR gymnasts, as both groups effectively reduced their use of negative self-talk from training to competition.

However, our results also revealed differences in the frequency of self-talk depending on the modality, particularly in training, where TR gymnasts used positive self-talk more frequently than their IR counterparts, demonstrating greater confidence and concentration. The increased use of positive self-talk among TR gymnasts during daily training could be linked to the shared distribution of responsibility and blame among teammates, a dynamic that is absent in individual sports (Alnuaimi et al., 2010). Sarig et al. (2023) similarly observed a higher frequency of spontaneous negative self-talk in individual sports compared to team sports, attributing this to the absence of social loafing in individual sports, where athletes bear the full burden of effort and responsibility.

When comparing self-talk behaviors between training and competition, further differences between the modalities were observed. While TR gymnasts maintained a consistent level of positive self-talk across both training and competition (with a slight decrease in anxiety control), IR gymnasts significantly increased their use of positive self-talk during competition. This behavior likely reflects their performance-oriented nature, given that the sample consisted of experienced gymnasts who had been competing from a young age. Such progressive learning and experience suggest that these athletes have developed strong coping mechanisms, allowing them to perceive competition as a challenge and adapt their self-talk to regulate confidence and concentration (Hatzigeorgiadis et al., 2011; Sarig et al., 2023). Consequently, although IR gymnasts used positive self-talk less frequently during daily training, they were able to spontaneously increase its usage in competitive situations.

1H4. was partly confirmed. While somatic and cognitive anxiety levels were similar between both groups, self-confidence was notably lower among individual rhythmic gymnasts. In aesthetic sports, where success is determined by judges, such as gymnastics or figure skating, higher anxiety levels have been reported. This is likely due to the pressure to distinguish oneself from competitors and the need for judges’ approval (Schaal et al., 2011). The lower self-confidence observed in IR gymnasts may be linked to a greater tendency towards avoidance behaviors, as individual athletes in stressful situations are unable to share blame or responsibility for their performance (Sarig et al., 2023). Clinically diagnosed anxiety was more prevalent in individual sports compared to team sports, which could be attributed to the social opportunities and stress relief provided by team sports, in contrast, the greater sense of isolation in individual sports might contribute to less healthy internal attribution following failure (Pluhar et al., 2019). Although our study focused on anxiety arising from competition situations, the cited evidence suggests that IR gymnasts may manage competitive anxiety less effectively than team rhythmic gymnasts. This is likely influenced by the usual training circumstances, such as lower levels of positive self-talk observed in IR athletes, which may affect their performance during competitions (Santos-Rosa et al., 2022).

The second objective focused on identifying the psychological variables that predicted precompetitive anxiety in both modalities. In summary, several significant associations were observed in individual gymnasts that were not present in team gymnasts, suggesting a greater sensitivity to precompetitive anxiety among individual athletes. Despite these differences, the predictor variables for competitive anxiety were consistent across both modalities. Specifically, positivity as a personality trait and negative self-talk during training were the only variables identified as predictors of precompetitive anxiety.

2H1. was not confirmed. The perception of an ego-involving motivational climate did not predict competitive anxiety in the rhythmic gymnasts analyzed, contrary to the evidence reported (Armada Martínez et al., 2021; Vazou et al., 2006). A larger sample size might yield more definitive results, particularly in TR gymnasts. The only significant association observed in individual gymnasts was intragroup rivalry, aligning with the emphasis placed on this aspect of the ego-oriented climate in individual sports (Castro-Sánchez et al., 2018). However, an unexpected negative association emerged, showing an indirect relationship between intragroup rivalry and both somatic and cognitive anxiety, as well as a direct but non-significant relationship with self-confidence. These results may reflect an adaptive mechanism among the individual gymnasts studied. Despite their age, these athletes had considerable competitive experience and were accustomed to comparing themselves with their opponents. Such experience may enable them to manage competitive stress more effectively, reducing levels of somatic and cognitive anxiety and enhancing self-confidence. Consequently, a major competition, such as qualifying for the national championship, may not be perceived as a loss of control or an unpredictable threat, thereby mitigating its potential to induce stress and its consequent anxiety (Cowden et al., 2014; Ntoumanis and Biddle, 1999).

2H2. was partly confirmed. Although negative associations were found between positivity and both somatic and cognitive anxiety, significant in individual gymnasts, positivity only predicted self-confidence in both modalities. Our findings align with previous research by Kemarat et al. (2022), which demonstrated that neuroticism, a trait characterized by the tendency to experience negative emotions, could predict competitive anxiety in athletes from both individual and team sports. As neuroticism is essentially the opposite of positivity, this parallel suggests a similar dynamic. Likewise, Armada Martínez et al. (2021) found a direct and significant relationship between pessimism and competitive anxiety in aesthetic gymnasts, while the relationship between optimism and anxiety was negative. Santos-Rosa et al. (2022) also observed direct relationships between positivity and positive self-talk, with indirect relationships when considering negative self-talk. They highlighted that higher levels of positivity were inversely related to somatic and cognitive anxiety and directly associated with self-confidence. However, in our study, positivity did not demonstrate predictive capacity for somatic and cognitive anxiety, likely due to the limited number of gymnasts in each group analyzed. Overall, the effect of positivity on anxiety can be explained from several perspectives. On the one hand, experience in competition and exposure to stressful stimuli may foster greater resilience in athletes with higher positivity, acting as a protective factor against anxiety. On the other hand, positive expectations for the future may enhance effort towards achieving goals (Armada Martínez et al., 2021).

2H3. was partly confirmed. Although associations were found between negative self-talk during competition and precompetitive anxiety, our results emphasized the predictive capacity of negative self-talk in a training context over that in competition for precompetitive anxiety. In IR gymnasts, negative self-talk predicted an increase in both somatic and cognitive anxiety, along with a decrease in self-confidence, while in TR gymnasts it only predicted cognitive anxiety. Previous research has reported the direct influence of precompetitive anxiety on negative self-talk during competition (Hatzigeorgiadis and Biddle, 2008), as well as the indirect relationship between negative self-talk and sports performance (Santos-Rosa et al., 2022; Van Raalte et al., 1994). The lower influence of negative self-talk during competition on precompetitive anxiety in our study may be attributed to the competition format in rhythmic gymnastics and similar sports that involve automated routines performed in front of a jury. At this point, it is important to consider characteristics of rhythmic gymnastics exercises, which last up to 90 seconds for IR and 150 seconds for TR gymnastics, featuring an automated and continuous sequence of choreographic elements, body movements, and apparatus handling (F.I.G., 2017). Given the competition experience of the selected gymnasts, it is reasonable to assume that during their routines, they exhibited high levels of concentration, confidence, and instructional (positive) self-talk, with low levels of negative self-talk. During competition, gymnasts are likely to focus entirely on executing their automated routines correctly, which contrasts with the different self-talk patterns observed in other sports, such as tennis, where athletes may experience more varied self-talk throughout a match lasting over two hours (Van Raalte et al., 1994).

On the other hand, positive self-talk during training was not associated with any precompetitive anxiety variables in TR gymnasts, although significant associations were observed in IR gymnasts. Specifically, IR gymnasts who frequently engaged in positive self-talk during training showed decreased cognitive anxiety and increased self-confidence before competition. This further highlights the differing behaviors between individual and team modalities. The anxiety experienced by IR gymnasts appeared more sensitive to the frequency of spontaneous self-talk, a finding consistent with previous studies (Georgakaki and Karakasidou, 2017). Akelaitis and Malinauskas (2018) found that individual athletes had less developed emotional skills compared to team athletes, particularly in self-awareness and self-regulation. The lower average values of positive self-talk in IR gymnasts, coupled with their lower emotional skills (Akelaitis and Malinauskas, 2018), may explain the indirect relationship between positive self-talk during training and cognitive anxiety in competition, as well as the direct relationship with self-confidence.

## Conclusions

The study highlighted that the interdependence level according to the rhythmic gymnastics' modality practiced (individual-independent or team-interdependent) significantly influenced the motivational climate, self-talk, and anxiety levels. Team gymnasts perceived a more ego-involved climate, characterized by intra-team rivalry, whereas both groups showed a greater perception of the task-involving climate versus the ego-involving climate. Team gymnasts used more positive self-talk during training, contributing to higher confidence and concentration. However, individual gymnasts increased their positive self-talk during competition, despite lower self-confidence. The role of positive personality in boosting precompetitive self-confidence was confirmed, including similar values in both modalities. The study identified that positive self-talk during training was associated with decreased cognitive anxiety and increased self-confidence in individual gymnasts, who demonstrated heightened sensitivity to self-talk’s effects. Negative self-talk, especially during training, was a predictor of precompetitive anxiety in both groups. The findings underscore the importance of regulating self-talk and managing rivalry in team gymnasts to improve team dynamics, while emphasizing the need for self-confidence and positive self-talk training in IR gymnasts. The study's insights can contribute to improving anxiety management strategies for athletes by tailoring interventions to the sport's specific interdependence level.
